# A Green Method for Bacterial Cellulose Electrospinning Using 1-Butyl-3-Methylimidazolium Acetate and γ-Valerolactone

**DOI:** 10.3390/polym17091162

**Published:** 2025-04-24

**Authors:** Elona Vasili, Bahareh Azimi, Mahendra P. Raut, David A. Gregory, Andrea Mele, Boyang Liu, Katrin Römhild, Marcus Krieg, Frederik Claeyssens, Patrizia Cinelli, Ipsita Roy, Maurizia Seggiani, Serena Danti

**Affiliations:** 1 Department of Civil and Industrial Engineering, University of Pisa, Largo Lucio Lazzarino 2, 56122 Pisa, Italypatrizia.cinelli@unipi.it (P.C.); 2 School of Chemical, Materials and Biological Engineering, Faculty of Engineering, University of Sheffield, Mappin st. S13JD Sheffield, Sheffield S10 2TN, UK; 3 Insigneo Institute for in Silico Medicine, Pam Liversidge Building, Sir Robert Hadfield Building, University of Sheffield, Sheffield S10 2TN, UK; 4 Thuringian Institute for Textile and Plastics Research (TITK), D-07407 Rudolstadt, Germany

**Keywords:** ionic liquids, cellulose dissolution, fibers

## Abstract

Bacterial cellulose (BC) is a highly pure and crystalline cellulose produced via bacterial fermentation. However, due to its chemical structure made of strong hydrogen bonds and its high molecular weight, BC can neither be melted nor dissolved by common solvents. Therefore, processing BC implies the use of very strong, often toxic and dangerous chemicals. In this study, we proved a green method to produce electrospun BC fibers by testing different ionic liquids (ILs), namely, 1-butyl-3-methylimidazolium acetate (BmimAc), 1-ethyl-3-methylimidazolium bis(trifluoromethylsulfonyl)imide (EmimTFSI) and 1-ethyl-3-methylimidazolium dicyanamide (EmimDCA), either individually or as binary mixtures. Moreover, γ-valerolactone (GVL) was tested as a co-solvent derived from renewable sources to replace dimethyl sulfoxide (DMSO), aimed at making the viscosity of the cellulose solutions suitable for electrospinning. A BmimAc and BmimAc/EmimTFSI (1:1 *w*/*w*) mixture could dissolve BC up to 3 w%. GVL was successfully applied in combination with BmimAc as an alternative to DMSO. By optimizing the electrospinning parameters, meshes of continuous BC fibers, with average diameters ~0.5 μm, were produced, showing well-defined pore structures and higher water absorption capacity than pristine BC. The results demonstrated that BC could be dissolved and electrospun via a BmimAc/GVL solvent system, obtaining ultrafine fibers with defined morphology, thus suggesting possible greener methods for cellulose processing.

## 1. Introduction

Cellulose, a linear polysaccharide composed of β-D-glucose units linked by β-1,4-glycosidic bonds, is the most abundant biopolymer on Earth. Its renewable nature, biodegradability in the environment and exceptional mechanical properties make it highly promising for sustainable material development [1]. However, its strong intermolecular and intramolecular hydrogen bonding contributes to its resistance to dissolution in most solvents [2]. Among the various forms of cellulose, bacterial cellulose (BC), produced through the microbial fermentation of various bacterial species, stands out for its exceptional purity and high crystallinity [2,3]. Indeed, BC exhibits superior mechanical strength and remarkable biocompatibility, making it highly desirable for applications in biomedical, electronic, industrial, food and agricultural fields [4,5,6]. Despite its several advantages, a widespread utilization of BC is hindered by its inherent properties, including high molecular weight, which impair its processing through common industrial methods [7]. To address this challenge, extensive research has focused on developing effective solvent systems for cellulose as well as BC dissolution [8]. Traditional methods involving alkali and acid-based treatments have shown promise, but often require prolonged treatment and pose relevant environmental concerns [9]. Common solvent systems such as NaOH/water, N,N-dimethylacetamide/lithium chloride (DMAc/LiCl) and N-methylmorpholine-N-oxide (NMMO) have been widely explored, yet they present drawbacks, such as incomplete dissolution, harsh processing conditions, toxicity concerns and environmental hazards [10,11]. For example, while DMAc/LiCl is a powerful cellulose solvent, it poses significant environmental and safety issues due to its toxicity and potential degradation of cellulose at temperatures > 85 °C [12,13]. Similarly, NMMO, though central to the Lyocell process, faces stability issues due to solvent and polymer oxidation at such temperatures [14,15,16].

In response to these challenges, ionic liquids (ILs) and deep eutectic solvents (DES) have emerged as promising alternatives to conventional solvents for cellulose [8,17]. ILs are organic salts with melting points below 100 °C, which appeared to be suitable for cellulose dissolution by offering tunable polarity and strong hydrogen-bonding capability under mild operating conditions. By carefully selecting specific cations and anions, ILs can be optimized to enhance their chemical and physical properties [10]. Similarly, DES, composed of hydrogen bond acceptors and donors, provide a cost-effective and environmentally friendly solution for BC pretreatment [18].

Recent studies have demonstrated that auxiliary techniques, such as ultrasonic- and microwave-assisted heating, can significantly increase the dissolution efficiency of IL and DES by reducing processing time and energy consumption. These methods facilitate the breakdown of BC intermolecular interactions, thereby accelerating dissolution and improving process sustainability [19]. Among them, microwave energy (MWE) enables rapid heating with lower energy input, making it a promising pretreatment technology [20]. However, while significant progress has been made in cellulose dissolution, translating these findings into functional material development still remains a key challenge.

Among the emerging techniques for cellulose processing, electrospinning stands out as a highly versatile method for transforming cellulose solutions into submicrometric fiber meshes with exceptional properties, including a high surface area-to-volume ratio, tunable porosity and high mechanical performance. By precisely adjusting key parameters, such as solution viscosity, conductivity and polymer concentration, electrospinning enables the fabrication of uniform ultrafine fibers with tailored structural and functional characteristics. Such fibers hold great potential for applications in tissue engineering, filtration, drug delivery and packaging. Compared to conventional fiber fabrication methods, electrospinning offers scalability and adaptability along with enhanced structural control, which overall suggests it to be a promising approach for developing advanced cellulose-based materials [21].

Building on these advancements, this study investigates the dissolution of BC, produced and characterized in three ILs, namely, 1-butyl-3-methylimidazolium acetate (BmimAc), 1-ethyl-3-methylimidazolium bis(trifluoromethylsulfonyl)imide (EmimTFSI) and 1-ethyl-3-methylimidazolium dicyanamide (EmimDCA), which were tested either in-dividually or as binary mixtures. By employing ultrasonic and MWE-heating, we aimed to enhance dissolution efficiency and reduce processing time. Following cellulose solubility testing, the most promising solvent system was selected for electrospinning to produce BC ultrafine fibers with tailored properties. Additionally, the potential of γ-Valerolactone (GVL) as a co-solvent, in place of the commonly used dimethyl sulfoxide (DMSO) was explored to improve solution viscosity and fiber formation during electrospinning. Electrospun BC fiber meshes were produced and characterized by morphology and water retention capacity. The preliminary findings obtained hold potential for several applications of BC ultrafine fibers in sensors, tissue engineering and biodegradable packaging.

## 2. Materials and Methods

### 2.1. Materials

*Komagataeibacter xylinus,* also known as *Gluconacetobacter xylinus* (NCIMB5346), was obtained from the Leibniz Institute DSMZ-German Collection of Microorganisms and Cell Cultures GmbH (Braunschweig, Germany). Hestrin–Schramm (HS) medium and Yeast–Peptone–Mannitol medium (YPM medium) for bacterial cultures were prepared in-house using the ingredients listed below. The yeast extract for culture medium preparation (code 70161) was purchased from Merk-Millipore (Darmstadt, Germany). Glucose (code 50-99-7), NaOH (code 1310-73-2) and Mannitol (code 4509) were acquired from MSK Ingredients Ltd. (Chesterfield, UK). HNa_2_O_4_P (anhydrous; code A11817) was bought from Alfa Aesar Chemicals (Ward Hill, MA, USA). Phosphate buffered saline (PBS), Peptone (code 70175), Bacteriological Agar (code A5306), Citric acid (code 27109), Acetic acid (glacial; code A6283, ≥ 99%), Acetone (code 270725, ≥ 99.9%), N,N-dimethylacetamide (DMAc; code 270555, ≥ 99.9%*),* Lithium Chloride (code 310468), BmimAc (code 39952, ≥95%), EmimTFSI (code 711691, ≥ 98%), EmimDCA (code 713384, ≥ 98%), DMSO (code 5.89569, 99.9%) and GVL (code 918660, ≥ 99%) were purchased form Sigma-Aldrich (Milan, Italy).

### 2.2. BC Production

BC was produced by bacterial fermentation under static conditions in the laboratory of Professor I. Roy, University of Sheffield, Sheffield, UK using HS medium [1,22], and modified YPM medium [23]. The composition of HS medium was as follows: yeast extract 5 g/L, peptone 5 g/L, glucose 20 g/L, disodium phosphate (anhydrous) 2.7 g/L and citric acid 1.15 g/L. The composition of modified YPM medium was as follows: yeast extract 5 g/L, peptone 25 g/L and mannitol 25 g/L. The initial pH was adjusted to 6.0 by HCl or NaOH in both media.

Inoculum was prepared in a 50 mL Falcon tube containing 10 mL of sterile HS medium by transferring a single colony of *K. xylinus* from a freshly prepared HS agar plate. This culture was incubated at 28 °C under static conditions for 3 days or until BC pellicle appeared at the liquid and air interface (i.e., primary inoculum). A 10% (*v*/*v*) primary inoculum was aseptically transferred into a 1 L Duran bottle containing 50 mL of fresh HS and modified YPM medium and incubated for 2 days (i.e., secondary inoculum). As a final step, 10% (*v*/*v*) of the secondary inoculum was used to inoculate 200 mL of HS and YPM medium in tissue culture trays (i.e., production stage). The lids of Falcon tubes, Duran bottles and trays were kept slightly loose to ensure enough oxygen flow throughout the fermentation period. Static fermentation was then carried out at 28 °C for 7 days.

The BC pellicles produced at the liquid–air interface during the fermentation process were manually harvested. After harvesting, the BC pellicles were placed on the sieve to allow excess liquid to drain away. Once the excess liquid had been removed, the BC pellicles were weighed. All pellicles were then washed three times with deionized water and resuspended in 0.1 M NaOH solution. The pellicles were purified at 80 °C for 1 h under constant stirring conditions. This process was repeated several times until the pellicles were free of medium impurities and bacterial cells. The pellicles were then further washed multiple times with double distilled (dd)-water until a neutral pH was reached. Therefore, they were either left hydrated, air-dried or freeze-dried and stored at room temperature (RT) until further analysis.

### 2.3. BC Characterization

The molecular weight (Mw) distribution of BC was determined using Size Exclusion Chromatography (SEC) equipped with a Mw-sensitive detector, providing absolute Mw values. Briefly, 2 g of BC pellicle was cut into small pieces. The sample was first allowed to swell in water overnight. Subsequently, water was sequentially replaced with acetone, followed by DMAc. After removing excess solvent and determining the solid content, approximately 0.01 g of BC was weighed into a volumetric flask and dissolved in 2.5 mL of DMAc containing 8% (*w*/*v*) LiCl. For SEC measurement, the resulting solution was further diluted with DMAc to achieve a final LiCl concentration of 0.8% (*w*/*v*) and then filtered through a 0.45 µm frit. A low-angle light-scattering detector (7°, LALS, λ = 670 nm) and a viscosity detector were used in combination with Mw-sensitive detectors to ensure accurate characterization of the BC Mw distribution. The results of interest were average molecular weight (Mw; g/mol); number average molecular weight (Mn; g/mol); z-average molecular weight (Mz; g/mol) and the polydispersity index (Mw/Mn). The equipment used for cellulose characterization was a Gel Permeations system Malvern-Panalytical, USA, which included a Refractometer (670 nm, RID 3580), a low angle light-scattering, a right angle light-scattering (90°, RALS, λ = 670 nm) and a 4-capillary-difference viscosimeter. All detectors and columns were maintained at 60 °C.

The morphology of as produced BC was analyzed via scanning electron microscopy (SEM) using a FEI Quanta 450 FEG (FEI, Hillsboro, OR, USA). The sample was previously dried for 24 h and subsequently coated with a thin layer of gold using a high-vacuum sputter and C-thread coater (Leica EM ACE600; Leica Microsystems, Wetzlar, Germany). This precise coating process enhanced sample conductivity and improved image quality by reducing charging effects.

### 2.4. Preparation of BC Solutions

The cryomilling technique was used to break down BC into fine particles or nanostructures while preserving its structural integrity and preventing thermal degradation. To cryomill BC, first, the liquid nitrogen tank was connected to the cryomill using a spanner to ensure a tight seal, then the valve was opened to a roughly 10-degree angle while monitoring the pressure to ensure it stayed above 1 bar. The grinding jar chamber was filled with the BC sample and milling ball, and the jar was inserted into the machine and sealed tightly. The plastic lid was closed, and the system was set to precool for 3 min to reach −150 °C and the sample was milled for 45 s at 25 Hz. Cryomilled BC was stored in an oven at 60 °C to remove any humidity. Three different methods were used for BC dissolution, as described hereafter:

Method 1. The specific concentration of BC solution preparation was performed using a stepwise procedure consisting of adding BC to the solvent under a magnetic stirrer for 8 h at 80 °C to reach homogeneity.

Method 2. As a second method, the dissolution of BC was performed using a CEM Discover 2.0 single-mode microwave reactor, which was equipped with a power source (up to 300 W), pressure control (up to 300 psi) and temperature regulation (up to 300 °C). BC was combined with the selected solvent in a 10 mL CEM microwave reaction vial and heated to 80 °C. The vial was fitted with a 10 mL optic-fiber accessory, which included a gas inlet for the controlled introduction of hydrogen gas into the reaction vessel. Temperature monitoring was conducted using a calibrated infrared temperature controller positioned beneath the optic-fiber probe, ensuring precise thermal regulation. A Teflon-coated magnetic stir bar was used to continuously stir the mixture at 600 revolutions per minute (rpm) during the process. Temperature, pressure and power profiles were tracked and recorded using manufacturer-provided software for real-time monitoring and analysis. The microwave synthesis program consisted of a 3 min preliminary pre-stirring stage, followed by a 20 min holding stage at 80 °C.

Method 3. The ultrasonic irradiation process was conducted as a third method for BC dissolution, using a Fisher Scientific ultrasonic bath (model FB-15051), which has a 2.75 L capacity, variable temperature control (up to 80 °C) and power output (up to 280 W) with a constant frequency of 37.0 kHz. BC dispersion was subjected to ultrasound irradiation for 3–6 h in the ultrasonic bath, as detailed in Table 1, operating at 37 kHz with a maximum ultrasonic peak power of 320 W and standard sine-wave modulation. To prevent excessive heating, the bath was connected to a cooling system, ensuring that the water bath temperature was maintained at 80 °C throughout the process, i.e., for 6 h.

### 2.5. Characterization of BC Solutions

The optical density (O.D.) test was employed to assess the dissolution behavior of BC in a colloidal solution by monitoring changes in color and absorbance. The initial indication of BC non-dissolution was observed through visible color changes upon its addition to the solution. To further evaluate dissolution, surface plasmon resonance (SPR) analysis was conducted by measuring the absorbance of the colloidal solution using a JANEWAY 6305 Spectrophotometer over a wavelength range of 190–1000 nm. This approach provided insights into the interaction between BC and the solvent, helping to determine the extent of dissolution and potential aggregation.

The selected solutions underwent rheological analysis. Steady shear viscosities were measured at RT using a Vibro Viscometer SV-1A (A&D, Company Limited, Tokyo, Japan). The instrument utilized two thin sensor plates arranged in a tuning fork configuration, driven to vibrate at their natural resonant frequency of 30 Hz by electromagnetic force. The viscosity was calculated based on the proportional relationship between the viscous resistance of the sample fluid and the amount of electric current required to drive and maintain the sensor plates at a constant vibration amplitude. Viscosity measurements were performed using 2 mL of liquid sample.

### 2.6. Electrospinning of BC Solutions

**Figure 1:**
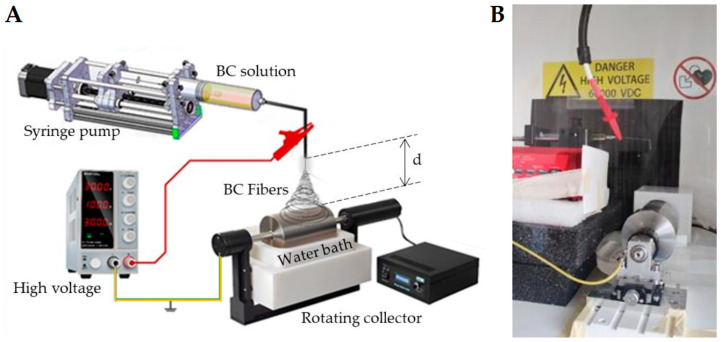
(**A**) Schematic of the electrospinning apparatus used to spin BC solutions, with d = distance from the needle tip to the collector surface. (**B**) Photograph of the spinning part of the used apparatus.

The solutions were spun using an electrospinning bench apparatus (Linari Engineering s.r.l., Pisa, Italy) equipped with a rotating collector and a static bath filled with dd-H_2_O (Figure 1). The static bath was positioned to be accessed by the rotating collector, which was set at a velocity of 50 rpm with a ground charge and placed at a distance *d* of 9 cm from the needle tip (21G × 3/4). The electrospinning process utilized a flow rate of 0.3 mL/h and a voltage range of 20–23 kV. After electrospinning was completed, the sample was left overnight in the coagulation bath filled with fresh d-water to remove all traces of the IL from the fibrous bacterial BC mesh.

### 2.7. Characterization of Electrospun BC Fibers

The morphology of the produced BC fibers was analyzed by a Phenom Pro Desktop SEM apparatus (Thermo Fisher Scientific; Milan, Italy), using the same procedure reported in Section 2.3 to ensure optimal visualization and structural analysis. Prior to observation, the samples were sputter-coated with gold with a S150B Sputter Coater by Edwards High Vacuum International (West Sussex, UK). Fiber size and pore size distributions, as well as fiber surface analysis of the electrospun meshes were obtained by employing the software “ImageJ” (NIH; Version 1.54p) by analyzing SEM micrographs. For fiber size, at least 100 different fibers were counted (*n* = 100). To estimate the surface area of fibrous structures observed in SEM images, a computational image analysis approach was applied. First, the pixel-to-micron scale was determined using the embedded scale bars. Fiber diameters were measured by detecting the edges and contours within the images and, assuming cylindrical geometry, the surface area per unit length (A*) was calculated using the formula given in Equation (1):

A* = πd_f_ (1)

where d_f_ is the fiber diameter.

To estimate the total fiber length visible in the image, we applied binarization and morphological skeletonization to reduce fibers to 1 pixel-wide paths and then converted the total pixel length to micrometers. The total fiber surface area was then obtained by multiplying the estimated total fiber length by the surface area per unit length (A*). This method for image-based estimation of fiber surface area is suitable for comparative analyses across different samples.

Fourier transform infrared spectroscopy (FTIR) (FT-IR Spectrum Spotlight 300, Perkin-Elmer, Springfield, IL, USA) was applied to study the conformational chemical characteristics of BC in a transmittance mode, by scanning a wavelength range from 4000 cm^−1^ to 400 cm^−1^. FTIR analysis provided information on the chemical structures and physical characteristics of the produced BC.

Absorption capacity was determined by immersing the dried electrospun meshes and BC samples in either dd-H_2_O or PBS at RT until equilibrium was reached. After immersion, the samples were removed from the water and excess surface water was blotted off with blotting paper. The weight of the swollen samples was then measured. This procedure was repeated at pre-established time intervals up to 24 h to ensure accuracy [24]. The absorption capacity (AC) of the samples was calculated using Equation (2):

AC (%) = (W_h_ − W_d_)/W_d_ × 100 (2)

where W_h_ is the weight of the hydrated samples, whereas W_d_ is the weight of dry samples.

## 3. Results

### 3.1. BC Characterization

**Figure 2:**
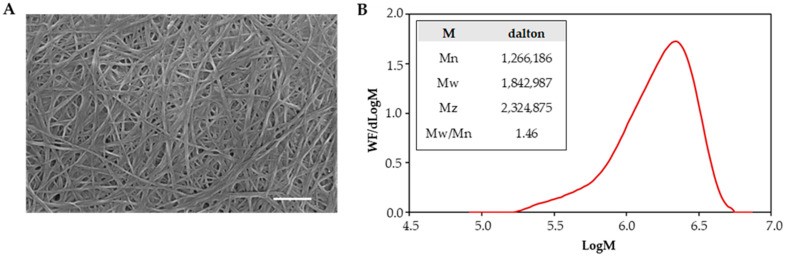
Characterization of BC as obtained from fermentation: (**A**) SEM micrograph of the fibrous structure of BC (10 kV, scale bar is 500 nm; magnification 100,000×), and (**B**) molecular weight distribution of BC; incorporated table showing number-average (Mn), weight-average (Mw), z-average (Mz) and the polydispersity index (Mw/Mn).

During the stationary cultivation of *Komagataeibacter xylinus*, BC was formed as a film on the surface of the nutrient medium. Figure 2A displays the SEM morphology of BC as produced, showing the presence of tightly arranged fibers with variable size, most of them in the tens-of-nanometer range. Figure 2B depicts the molecular weight distribution of the obtained BC, which is further detailed in the inset table according to number-average (Mn), weight-average (Mw), and z-average (Mz) molecular weights of BC, determined by SEC analysis.

### 3.2. BC Dissolution and BC Solution Characterization

Table 2 summarized the outcomes of BC dissolution in the tested solvent systems. BC dissolved completely at the investigated concentrations (0.5–3.0% *w*/*w*) in BmimAc using various assisted techniques such as microwaves, ultrasonic bath or simple mixing under the applied time and temperature conditions. Among them, microwave heating significantly accelerated dissolution, reducing the dissolution time from 8 h to just 20 min, while ultrasonic treatment shortened the dissolution time from 8 h to 6 h.

**Figure 3:**
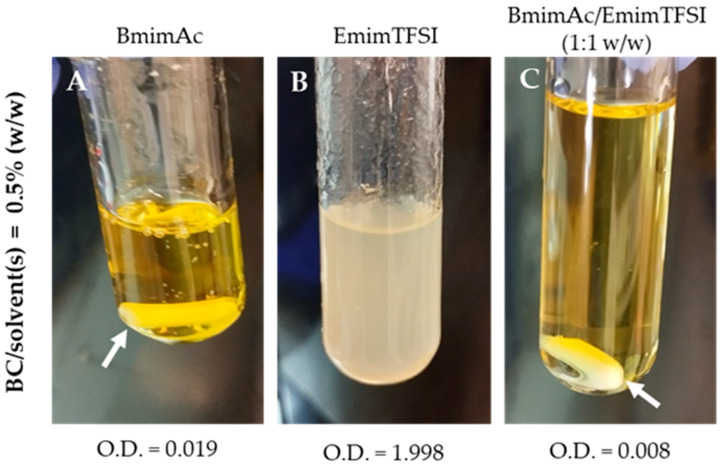
Dissolution of BC in different solvent systems at 0.5% *w*/*v* concentration and respective optical densities (O.D.): (**A**) BmimAc, (**B**) EmimTFSI and (**C**) BmimAc/EmimTSFI (1:1 *w*/*w*). Arrows indicate a magnet, left inside to show transparency.

The O.D. results further confirmed the dissolving capabilities of the tested systems. When cellulose is not fully dissolved, the medium becomes turbid, indicating incomplete dissolution. Consequently, O.D. serves as an effective parameter for measuring cellulose dissolution in a solvent: the lower O.D., the higher cellulose solubility [24]. The BmimAc/EmimTFSI (1:1 *w*/*w*) mixture exhibited the lowest O.D. values, indicating the highest degree of BC dissolution compared to BmimAc and EmimTFSI, individually. To provide a visual comparison, Figure 3 shows the photographs of BC solutions at a concentration of 0.5% (*w*/*w*) in three different solvent systems and indicates the corresponding O.D. After identifying BmimAc and the BmimAc/EmimTFSI (1:1, *w*/*w*) mixture as effective solvent systems for BC dissolution, the viscosity of the resulting solutions at various concentrations was analyzed to assess their suitability for the electrospinning process.

Table 3 reports the viscosity values for the selected solvent systems at varying BC concentrations. BC/IL solutions at 3.0% (*w*/*w*) showed a solid-like behavior and their viscosity could not be assessed. Therefore, for concentrations of 3% (*w*/*w*), i.e., the most concentrated ones, the addition of a co-solvent was needed to reduce their viscosity.

### 3.3. BC Solution Electrospinning and Characterization of Electrospun BC Fibers

**Figure 4:**
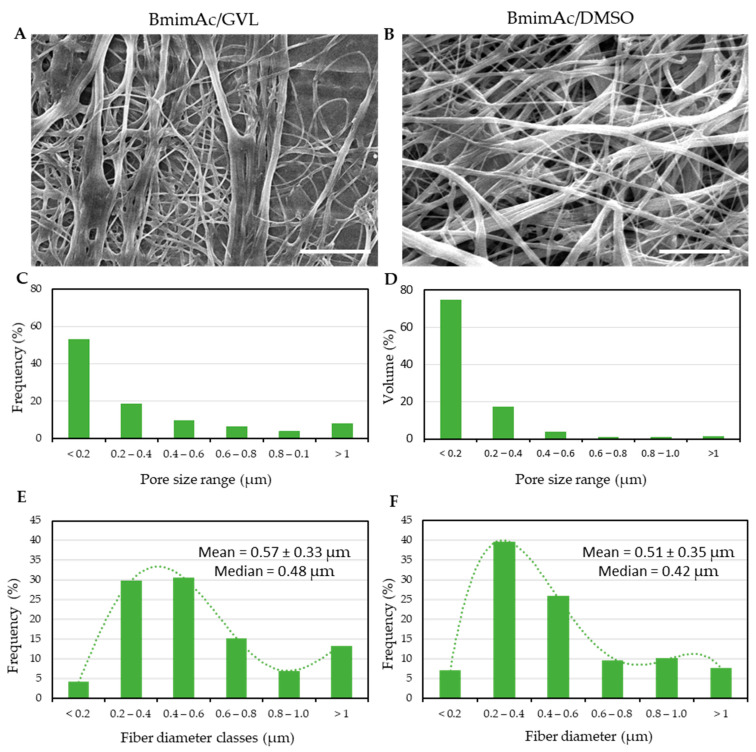
Morphological analysis of electrospun BC (3.0% *w*/*w*) in (**A**,**C**,**E**) BmimAc/GVL and (**B**,**D**,**F**) BmimAc/DMSO. (**A**,**B**) SEM micrographs showing the fibers obtained by dissolving BC in (**A**) BmimAc/GVL and (**B**) BmimAc/DMSO (15 kV, 4000× magnification, scale bar is 10 µm). (**C**,**D**) Bar graphs displaying the void volume distribution into pores discriminated by size ranges for BC dissolved in (**C**) BmimAc/GVL and (**D**) BmimAc/DMSO. (**E**,**F**) BC fiber diameter distribution into pore size classes, electrospun using (**E**) BmimAc/GVL and (**D**) BmimAc/DMSO. Mean ± standard deviation and median are reported for BmimAc/GVL and (**D**) BmimAc/DMSO; a qualitative trend of the distributions is obtained using the 5th and 4th order polynomial equations, respectively.

The results of fiber morphological characterization are shown in Figure 4.

Among the solutions listed in Table 3, only BC/IL solutions up to 1.0% (*w*/*w*) could be loaded in the syringe and tested via electrospinning; however, they did not lead to the formation of continuous fibers at varying flow ratios, voltage as well as needle-to-tip distance. The addition of DMSO or GVL as co-solvents allowed BC solutions with increased concentration, i.e., 3.0% (*w*/*w*), to be successfully electrospun into continuous fibers. The morphology and pore size analysis of the successfully electrospun BC meshes are given in Figure 4. Figure 4A,B shows SEM micrographs of electrospun fibers obtained by employing a rotating collector partially submerged in a distilled water bath, operating at a rotational speed of 50 rpm. This set-up facilitated solvent removal during the fiber formation. Pore size distribution analysis (Figure 4C,D) displayed similar trends of void volume, which is more frequently represented for pores with an equivalent diameter lower than 0.4 µm. Among the two solvent systems used, BmimAc/DMSO led to a lower frequency of larger pores. The outcomes of fiber diameter distribution are given in Figure 4E,F. Fiber sizes were found to be in a similar range (mean: 0.57 ± 0.33 µm and 0.48 µm median for BmimAc/GVL; mean: 0.51 ± 0.35 µm and 0.42 µm median for BmimAc/DMSO); however, some differences could be outlined by using the two solvent systems. In both cases, the fiber size distributions were positively skewed, since the mean values exceeded the median values, thus indicating that most of the fibers were thinner, with occasionally thicker fibers than mean values.

**Figure 5:**
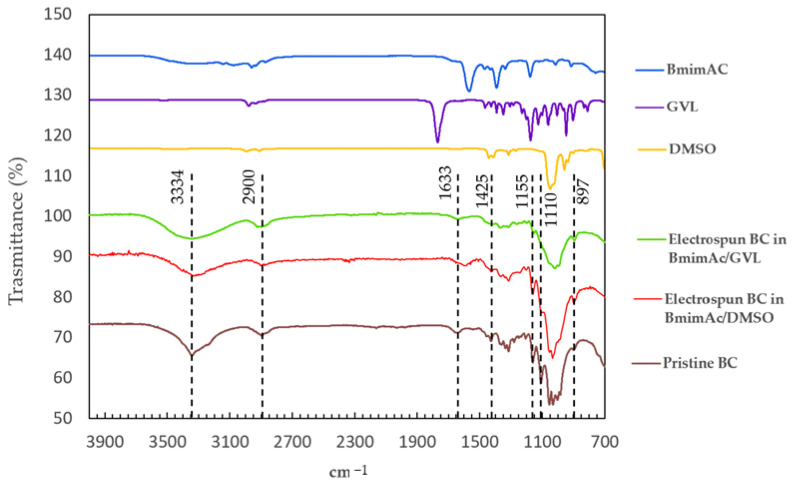
FTIR spectra of electrospun BC fibers obtained from BmimAc solutions added with either GVL or DMSO as co-solvents, compared with pristine BC, solvent (BmimAC) and co-solvents alone.

FTIR analysis was performed on pristine BC and electrospun BC fibers produced with different solvent systems (Figure 5).

All samples were characterized using FTIR in ATR mode. The electrospun BC fibers exhibited similar characteristic features to those of pristine BC, without the appearance of any new peaks. The characteristic bands of BC were observed in the spectra of the BC fibers, including the following characteristic peaks: 3334 cm^−1^ attributable to O–H stretching of cellulose I hydroxyl groups; 2884 cm^−1^ attributable to CH_2_ asymmetric stretching; 1155 cm^−1^ attributable to asymmetric stretching of C–O–C and CH deformation; 892 cm^−1^ attributable to CH out-of-plane bending vibration) and 1110 cm^−1^ (assigned to C–O–C stretching within an anhydroglucose ring) [25]. The relative intensity between the absorption band at 1420–1430 cm^−1^, typically associated with CH_2_ scissoring vibrations of crystalline cellulose I, and the band around 897 cm^−1^, attributed to C–H bending vibrations in amorphous regions, are commonly used as an indicator of cellulose crystallinity [26]. In the electrospun BC fibers, a decreased intensity of the 1420–1430 cm^−1^ band relative to the 897 cm^−1^ band was observed, which is attributed to lower crystallinity regions [27]. In addition, a comparison of the FTIR spectra of native BC and electrospun BC fibers with those of the solvents used revealed that the characteristic solvent peaks are absent in the BC samples. This indicates that the washing step with deionized water effectively removed all solvent residues from the fibrous bacterial cellulose mesh.

The surface area of pristine BC and electrospun BC (using BmimAc/DMSO) was conducted via ImageJ analysis of SEM micrographs. By comparing the calculated fiber surface area normalized by the surface area of the samples in pristine BC and electrospun BC fiber meshes, we obtained a slightly higher value in pristine BC (i.e., BC/electrospun BC area = 1.16), which is in agreement with the smaller fiber size in this sample.

**Figure 6:**
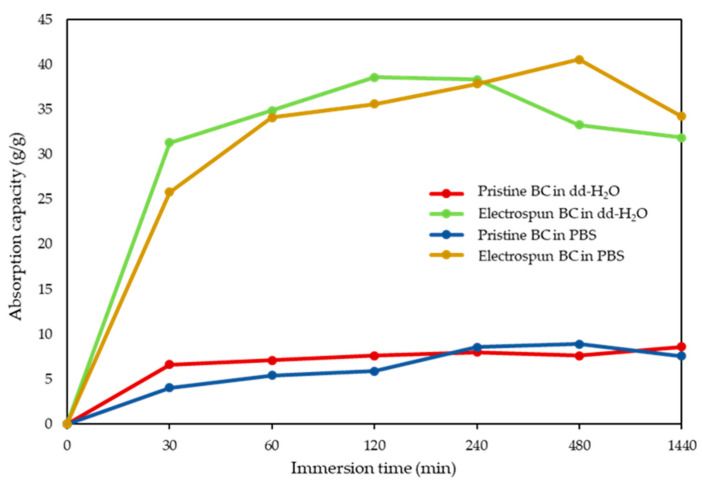
Graph showing the absorption capacity trends during the soaking time of pristine BC and electrospun BC fibers [i.e., 3% (*w*/*w*) BC in BmimAc/DMSO], immersed in dd-water and PBS media.

The electrospun BC meshes demonstrated higher absorption capacity both in dd-H_2_O and in PBS with respect to pristine BC, as shown in Figure 6.

## 4. Discussion

BC is an appealing source of pure cellulose, which can be produced via fermentative processes, considered to be a sustainable cellulose production method for this extraordinary biopolymer. Due to its chemistry, high molecular weight and crystallinity, BC suffers from the same and even higher processability limitations of cellulose, namely, the need for strong and impactful solvents for its dissolution, since cellulose cannot be processed from the molten state. The search for greener solvents than those currently utilized for cellulose and BC has thus become essential.

In this study, the dissolution behavior of BC in different ILs, using ultrasonic and microwave-assisted heating to enhance dissolution efficiency, was investigated in view of obtaining BC solutions suitable for electrospinning. In fact, cellulose is industrially used within dry–wet spinning processes to obtain fibers (i.e., Lyocell process) and, recently, BC is attracting interest in the biomedical field, where electrospinning is widely applied to produce scaffolds and patches [28]. Therefore, having proof-of-concepts of BC spinning in green solvents could improve the sustainability and biocompatibility of the obtained products.

At first, BC was synthesized from *Komagataeibacter xylinus* under stationary conditions, and BC samples, consisting of tightly arranged ultrafine fibers produced by the bacteria with narrowly distributed molecular weight (Mw = 1.8·10^6^, polydispersity index = 1.46), were formed. In the subsequent phase, the dissolution behavior of the synthesized BC was evaluated using BmimAc, EmimDCA and EmimTFSI at varying concentrations and through different processing methods. BC was dissolved completely in BmimAc across various concentrations and heating techniques. Notably, microwave heating significantly accelerated BC dissolution, reducing the processing time from 8 h (as by stirring) to 20 min, whereas ultrasonic treatment shortened the dissolution time to 6 h. This improvement is attributed to the ability of microwaves to interact directly with polar molecules and ionic solvents, thereby enhancing ionic mobility, mass transfer and solvent penetration into the cellulose structure. These combined effects demonstrated the effectiveness of microwave heating in dissolving BC, making it a highly efficient approach for cellulose processing [29,30].

Cellulose solvents can be broadly categorized into derivatizing and non-derivatizing types. Derivatizing solvents chemically modify cellulose before dissolution (e.g., esters, ethers and acetals), whereas non-derivatizing solvents disrupt hydrogen bonds directly without altering cellulose chemistry, which results in better outcomes and higher mechanical strength for cellulose products. As part of their nature, ILs can interact with hydrogen bonds of cellulose. The BC solubility differences among the tested ILs can be attributed to the nature of their anions. The acetate anion (Ac^−^) in BmimAc is a strong hydrogen bond acceptor, effectively disrupting the extensive hydrogen bonding network within BC, thereby facilitating dissolution. In contrast, the dicyanamide (DCA^−^) and bis(trifluoromethylsulfonyl)imide (TFSI^−^) anions exhibit significantly weaker hydrogen bonding capabilities, making them less effective in breaking the crystalline structure of cellulose. Additionally, higher basicity and strong interaction of acetate with the hydroxyl groups in BC further contribute to its excellent solubilization performance. Consequently, while BmimAc efficiently dissolved BC, EmimDCA and EmimTFSI failed to provide the necessary interactions, resulting in non-dissolution. Mixing two or more ILs is known to represent a promising way for designing solvent systems with tailored properties. In this context, BmimAc was mixed with EmimTFSI and EmimDCA to explore possible synergies. As shown, the mixture of BmimAc with EmimDCA was not effective in dissolving BC. This can be attributed to EmimDCA inability to disrupt hydrogen bonding and cellulose crystallinity to a sufficient extent. The acetate anion in BmimAc provides some degree of disruption, but without the strong complementary interactions derived from EmimDCA, the dissolution process remained incomplete [31].

On the other hand, the complete dissolution of BC in the BmimAc/EmimTFSI (1:1 *w*/*w*) mixture can be attributed to the synergistic interactions between the two ILs, which create a better effective dissolution environment than each IL alone. BmimAc disrupts cellulose hydrogen bonds, while TFSI^−^ stabilizes the dissolved cellulose, further reducing the solvent viscosity at the same BC content. This reduction in solvent system viscosity may improve BC penetration and facilitate efficient mass transfer. Additionally, the presence of TFSI^−^ can alter the polarity and ionic environment of the mixture, thus preventing the reaggregation of dissolved BC chains. This synergistic effect appeared to enhance cellulose solubility, making the BmimAc/EmimTFSI mixture a highly effective solvent system for BC dissolution [31].

After identifying BmimAc and the BmimAc/EmimTFSI (1:1, *w*/*w*) mixture as effective solvent systems for BC dissolution, the viscosity of the resulting cellulose solutions at various concentrations was analyzed to assess their suitability for the electrospinning process. Viscosity serves as a key indicator of solvent efficiency, as low viscosity reflects better cellulose dissolution and, consequently, improved polymer dispersion [32,33]. As reported, the use of BC solutions at concentrations of 0.5% (*w*/*w*) and 1% (*w*/*w*) in both solvents resulted in low-viscosity solutions, with values ranging in 350–1100 mPa·s for BmimAc and 272–602 mPa·s for the BmimAc/EmimTFSI (1:1 *w*/*w*) mixture, respectively. In both cases, the viscosity of the solution prepared with BmimAc/EmimTFSI (1:1 *w*/*w*) was lower than that of the solution produced with BmimAc, indicating a more efficient dissolution in the binary solvent system. Increasing the concentration up to 2.5% (*w*/*w*) led to a viscosity increase, although it remained insufficient for electrospinning fibers. However, at a 3% (w/w) BC concentration, the viscosity became too high to be measured, likely due to cellulose chain entanglement within the ILs [34]. Consequently, the use of co-solvents could provide a solution to this issue by reducing viscosity and enhancing spinning property. We demonstrated that the addition of DMSO and GVL as co-solvents significantly decreased the solution viscosity, making the 3% (w/w) BC solutions finally processable by electrospinning.

Electrospinning is an efficient technique for obtaining continuous nanofibers, allowing for precise control over the fiber size, orientation and the final mesh porosity. The fermentation process does not allow control over these parameters for BC fibers, so treatment via electrospinning offers better control over these properties [30]. BmimAc is recognized as one of the most effective solvents for dissolving both cellulose and BC to produce solutions suitable for electrospinning. Although BC dissolved well in BmimAc, the high viscosity of the solution made it difficult to control the electrospinning process and obtain continuous fiber production. Although polymeric solution jets, once set, tend to remain stable, bending instability may sometimes be experienced due to the electric field. When the viscosity of the solution is not appropriate, the polymer jet breaks into droplets instead of forming continuous fibers, leading to the formation of beads and particles rather than fibers [35]. Typically, as the viscosity of a cellulose solution increases, a greater overlap or entanglement of the cellulose chains occurs, facilitating jet elongation and preventing the solution from breaking up to a critical point, known as capillary instability. However, if the viscosity becomes too high, capillary instability can be suppressed, as observed with BC solutions dissolved solely in BmimAc. Another issue is the low electrical conductivity of ILs, which can distort the electric field and compromise control over fiber formation.

To overcome these challenges, several approaches have been explored, including adjusting solution concentrations, incorporating co-solvents or surfactants, optimizing electrospinning parameters and exploring alternative solvents or processing techniques to improve cellulose electrospinning ability in ILs [36]. DMSO can be employed as a co-solvent to regulate solution viscosity, reduce system entanglement density, improve electrical conductivity, facilitate better spinning of the solution and ultimately enhance solvent removal efficiency. Our results revealed that the BC solution containing DMSO exhibited typical shear-thinning behavior at high shear rates [30]. However, due to the toxicity of DMSO, GVL, obtained from renewable sources, such as lignocellulosic biomass (e.g., furfural and levulinic acid derived from agricultural waste) was tested as a co-solvent due to its recognized low toxicity. GVL also has a low vapor pressure and a high flash point, making it a safer alternative to organic solvents [37]. Using a 3% (*w*/*w*) BC solution, the BmimAc/GVL (1:3 *w*/*w*) mixture achieved a viscosity comparable to that of the BmimAc/DMSO (1:3 *w*/*w*) system, demonstrating its usefulness as a sustainable alternative to DMSO for electrospinning. To address the issue of solvent solutions not evaporating between the electrospinning spinneret and the collector, a custom-designed spinning procedure was implemented. In this method, BC fibers were deposited in a coagulant bath containing dd-H_2_O to remove the IL.

The set-up worked successfully and made the obtainment of BC electrospun fibers of similar size possible from both BmimAc/GVL and BmimAc/DMSO solutions (averagely, 520 nm and 570 nm, respectively). The fibrous meshes were also characterized in terms of pore features. Pore size distribution in cellulose fibers is a crucial factor that influences their performance in various applications [38]. The pore size distribution refers to the range and frequency of pores within the fibrous network. The results of pore size distribution measured on SEM micrographs revealed a similar trend for pore size, with the presence of smaller pores (≤ 0.2 µm) in both samples, mostly using DMSO as a co-solvent, and the presence of larger pores (> 1 µm), particularly using GVL as a co-solvent.

The fiber diameter distributions of electrospun bacterial cellulose (3% *w*/*w*) differed depending on the co-solvent system used with BmimAc. Samples prepared using GVL as a co-solvent (i.e., BmimAc/GVL, 1:3 *w*/*w*), exhibited a mean fiber diameter of 0.57 ± 0.33 µm and a median of 0.48 µm. In contrast, samples prepared with DMSO (i.e., BmimAc/DMSO, 1:3 *w*/*w*), showed a slightly lower mean diameter of 0.51 ± 0.35 µm and a median of 0.42 µm. In both cases, the mean exceeded the median, indicating positively skewed distributions likely caused by a minority of thicker fibers. The similarity in standard deviations suggested comparable variability across both systems. The moderate differences may reflect the influence of solvent properties on jet stability and fiber formation during electrospinning. It is known that DMSO is a polar aprotic solvent with a relatively high dielectric constant (~46.7). It can partially dissociate ionic interactions in BmimAc, increasing the overall charge stabilization of the BC solution. Differently, GVL is a cyclic ester with lower polarity compared to DMSO, leading to weaker charge repulsion. The different Z-potentials of BC solution in the two solvent systems may have ultimately resulted in slightly thicker fibers using GVL.

The surface area analysis indicated that the exposed surface area of pristine BC was about 1.16 times higher than that of electrospun BC fiber meshes. This is in agreement with the fiber size of raw BC (in the order of 10 nm), which imparts higher surface area-to-volume ratio than in electrospun BC (~500 nm). The value calculated via image analysis refers to the top layer of the samples, but it would largely increase considering the sample volume, since about 50× fiber layers would be found in pristine BC for each fiber layer of electrospun BC, when comparing the same volumes of samples. However, our study shows that BC fibers had a much higher absorption capacity of both deionized and salty water solutions (i.e., dd-H_2_O and PBS) than pristine BC, allowing them to retain up to 40 times their dry weight in water [39]. This capacity makes them a valuable material for applications spanning from medicine to environmental management. In medical and wound care applications, the ability of BC patches to absorb and retain large amounts of moisture is particularly advantageous, since maintaining a humid environment is essential for the optimal healing of certain wounds [40]. The remarkable difference in absorption capacity which was observed between raw and electrospun BC can be attributed to several factors, such as the diverse crystallinity occurred as a consequence of IL dissolution and the large pore size of electrospun BC fiber meshes. BC produced through fermentation generally exists as polymorph type I (i.e., cellulose I), showing a high amount of intermolecular and intramolecular hydrogen bonds, which entitle BC with a high crystallinity degree. In fact, the tightly packed crystalline regions are less accessible to water molecules, limiting the overall absorption capacity. When BC is dissolved in ILs, crystalline cellulose I transforms into polymorph type II (i.e., cellulose II) [41]. Indeed, during the dissolution process, IL molecules penetrate in between the hydrogen-bonded sheets of cellulose I, causing slight expansion of the fibrillar structure. DMSO also interferes with the formation of intermolecular hydrogen bonds, reducing the overall crystallinity compared to the starting BC [42]. Moreover, electrospinning BC solutions in ILs leads to a further increase in amorphous phases, contributing to the transition to cellulose II [43]. These amorphous regions are more accessible to water molecules, enhancing the fiber capability of absorbing and retaining water. The crystallinity index (CrI) of the electrospun BC in BmimAc/DMSO (CrI = 58.8%), as assessed in previous studies and in agreement with the literature data, has been found to be lower than the BC harvested from the bacteria (CrI = 85.5%), indicating that a crystalline polymorph transformation occurred from cellulose I to cellulose II, the latter containing both highly ordered (crystalline) and less ordered (i.e., amorphous) regions [28]. In the electrospun BC fibers, the decreased intensity of the 1420–1430 cm^−1^ band relative to the 897 cm^−1^ band observed, suggested a lower degree of crystallinity compared to the native BC structure. This behavior is consistent with previous studies reporting a reduction in crystallinity following electrospinning of regenerated cellulose [27]. This decrease in crystallinity can be attributed to the disruption of the native hydrogen-bonding network during BC dissolution, followed by rapid solvent removal and fiber solidification during electrospinning and coagulation, which hinder the reformation of an ordered crystalline structure.

The electrospinning process produced ultrafine fibers with a high surface-area-to-volume ratio and a highly porous structure, with both synergistically contributing to water absorption capacity. Considering the obtained findings, the lower crystallinity and greater pore size of electrospun BC demonstrated that they play a dominant role over a larger surface area of pristine BC in water absorption capacity.

This study shows the possibility of BC processing into fibers using electrospinning with a greener solvent system than conventional ones, which can enable a control of the rheological properties, namely BmimAc/GVL. This achievement may pave the way for further advancement in obtaining cellulose fiber-based products with increased sustainability in terms of materials used.

## 5. Conclusions

This study investigated the dissolution of BC in various ILs, both individually and in binary mixtures, using ultrasonic- and microwave-assisted heating to enhance efficiency. Notably, BmimAc and a BmimAc/EmimTFSI 1:1 (*w*/*w*) binary system were particularly effective in dissolving BC, leveraging the IL capacity to disrupt hydrogen bonds in the cellulose structure. Microwave-assisted heating significantly accelerated dissolution, reducing the duration by 40 times. We optimized the best solutions and the set-up for electrospinning, the latter by adding a coagulation bath to remove the non-volatile solvents. Due to their high viscosity, a co-solvent was necessary. GVL, derived from renewable sources, successfully replaced DMSO in this regard. Such a replacement can minimize the environmental and health risks associated with using DMSO. Electrospinning BC solutions in ILs, combined with DMSO or GVL, produced continuous ultrafine fibers with well-defined pore structures and improved absorption capacity both in water and in PBS with respect to raw BC. These characteristics indicate a significant potential of electrospun BC meshes as absorbent pads, useful for meat packaging and cosmetic applications, as well as restorative patches for exudative wounds, essential in advanced biomedical products.

## Figures and Tables

**Table 1:** The different solvent systems and concentrations of BC; dissolution was tested via 3 different methods: (method 1) stirring for 8 h at 80 °C; (method 2) microwave for 20 min at 80 °C, and (method 3) ultrasonic bath for 6 h at 80 °C.

Solvent/s	Solvent Ratio (*w*/*w*)	BC Concentrations (*w*/*w*%)
BmimAc	1	0.1, 0.5, 1.0, 2.5, 3.0
EmimDCA	1	0.1, 0.5, 1.0
EmimTFSI	1	0.1, 0.5, 1.0
BmimAc/EmimDCA	1:1	0.1, 0.5, 1.0
BmimAc/EmimTFSI	1:1	0.1, 0.5, 1.0, 2.5, 3.0
BmimAc/DMSO	1:3	0.1, 0.5, 1.0, 2.5, 3.0
BmimAc/GVL	1:3	0.1, 0.5, 1.0, 2.5, 3.0
BmimAc/EmimTFSI/GVL	1:1:6	0.1, 0.5, 1.0

**Table 2:** Dissolution results of BC in the different solvent systems and different BC concentrations via 3 different methods: (1) stirring (8 h at 80 °C); (2) microwave (20 min at 80 °C) and (3) ultrasonic bath (6 h at 80 °C).

Solvent/s (*w*/*w*)	BC Concentration (*w*/*w*%)	Microwave	Ultrasonic Bath	Stirring
BmimAc	0.1, 0.5, 1.0, 2.5, 3.0	Complete	Complete	Complete
EmimDCA	0.1, 0.5, 1.0	Poor	Poor	Poor
EmimTFSI	0.1, 0.5, 1.0	Poor	Poor	Poor
BmimAc/EmimDCA (1:1)	0.1, 0.5, 1.0	Poor	Poor	Poor
BmimAc/EmimTFSI (1:1)	0.1, 0.5, 1.0, 2.5, 3.0	Complete	Complete	Complete
BmimAc/DMSO (1:3)	0.1, 0.5, 1.0, 2.5, 3.0	Complete	Complete	Complete
BmimAc/GVL (1:3)	0.1, 0.5, 1.0, 2.5, 3.0	Complete	Complete	Complete
BmimAc/EmimTFSI/GVL (1:1:6)	0.1, 0.5, 1.0	Complete	Complete	Complete

**Table 3:** Room temperature (RT) viscosity of BC solutions at varying BC concentrations.

Solvent Systems (*w*/*w*)	BC (*w*/*w*%)	Viscosity (mPa·s)
BmimAc	0.0	243
	0.5	359
	1.0	1100
	3.0	Too viscose
(BmimAc/EmimTFSI) (1:1)	0.0	190
	0.5	272
	1.0	602
	3.0	Too viscose
BmimAc/DMSO (1:3)	3.0	5847
BmimAc/GVL (1:3)	3.0	4735

## Data Availability

The data that support the findings of this study are available from the corresponding authors upon reasonable request. The data are not publicly available due to [because the study is part of ongoing research and data collection is not yet complete].
